# Pediatric Intestinal Failure Review

**DOI:** 10.3390/children5070100

**Published:** 2018-07-20

**Authors:** Nisha Mangalat, Jeffrey Teckman

**Affiliations:** 1 Division of Gastroenterology, Department of Pediatrics, Cardinal Glennon Children’s Hospital, Saint Louis University, Saint Louis, MO 63104, USA; nisha.mangalat@health.slu.edu; 2 Department of Biochemistry and Molecular Biology, Saint Louis University, Saint Louis, MO 63104, USA

**Keywords:** pediatric intestinal failure, short bowel syndrome, parenteral nutrition, intestinal failure associated liver disease, enteral nutrition, ethanol lock, catheter related blood stream infections

## Abstract

The term, ‘intestinal failure’, signifies the inability of the body to meet the digestive, absorptive and nutritive needs of the body. As such, these individuals require parenteral nutrition (PN) for survival. The subsequent nutritional, medical and surgical facets to the care are complex. Improved care has resulted in decreased need for intestinal transplantation. This review will examine the unique etiologies and management strategies in pediatric patients with intestinal failure.

## 1. Introduction

The term ‘intestinal failure’ is widely defined as a “reduction in functional gut mass below the minimal necessary for digestion and absorption adequate to satisfy the nutrient and fluid requirements for maintenance in adults or growth in children” [1]. Intrinsic to the definition, these patients absolutely depend on parenteral nutrition (PN) for survival.

Intestinal failure (IF) is generally categorized as occurring secondary to (1) short bowel syndrome, referring to the spectrum of malabsorption that occurs after reduction of mucosal surface area from congenital or acquired lesions; (2) dysmotility (intestinal pseudoobstruction); or (3) mucosal enteropathy (e.g., congenital chloride diarrhea, tufting enteropathy, and microvillus inclusion disease). Table 1 lists the most common causes of pediatric intestinal failure. In some cases, patients have features of a few or all the categories of IF (i.e., gastroschisis). In children, the most common cause of IF is short bowel syndrome [2].

Short bowel syndrome is characterized by a lack absorptive capacity due to severely reduced mucosal surface area. Necrotizing enterocolitis is the leading cause of pediatric SBS and subsequently the most common cause of pediatric intestinal failure in developed countries [3]. Despite advances in the care of the neonate, the incidence of NEC has remained relatively stable over the previous decades. The Eunice Kennedy Shriver National Institute of Child Health and Human Development Neonatal Research Network (NRN), a national consortium representing 69 academic medical centers in the United States, reported mean incidence of NEC of 3% to 11% between 1997 and 2000 [4] and 5% to 15% between 2003 and 2007 [5]. 

Another important cause of pediatric SBS is gastroschisis, and recent data from the CDC indicates that the prevalence is increased by 30% from 3.6 per 10,000 births during 1995–2005 to 4.9 per 10,000 births during 2006–2012. The reasons for this increase are unclear and subject for further study [6]. Other etiologies of SBS include mid gut volvulus, and long segment Hirschsprung’s disease [7,8].

## 2. Goals of Therapy

In 1968, Dudrick and Willmore first reported the successful management of neonatal short bowel syndrome using an intravenous nutrition strategy [8]. Before this time, the diagnosis of SBS carried a uniformly grave prognosis. While PN is invariably a lifesaving management strategy in the care of IF patients, there is inherent morbidity and mortality related to its use, primarily in the form of liver disease and sepsis. The overall goal in the management of patients with IF is independence from parenteral nutrition, or ‘enteral autonomy’.

Enteral autonomy occurs through intestinal adaptation, the process by which the intestine compensates to massive bowel resection to improve nutrient and fluid absorption in the remaining bowel. There are demonstrable changes in intestinal morphology after resection including increased cellular proliferation and angiogenesis [9,10]. These changes enhance mucosal growth and may enhance absorptive capacities [11]. Animal studies have shown intestinal resection is associated with increased expression of transporter proteins critically important in nutrient, electrolyte and water absorption (sodium glucose, Na/H exchangers, and Na/K adenosine triphophatases), even independent of increases in enterocyte mass [12].

Multiple factors influence the extent of intestinal adaptation including age at time of injury; length and integrity of residual bowel; presence ileocecal valve; the presence of an intact colon in continuity, composition, timing, and advancement of enteral feedings; luminal environment (absence of small bowel bacterial overgrowth); absence of severe liver disease and normal GI motility [13,14]. Plasma citrulline level has been utilized as a biomarker of enterocyte mass, and a potential predictor of intestinal failure outcomes. In a cohort of patients with short bowel syndrome due to surgical resections, a plasma citrulline level of <20 mMol/L had a 95% positive predictive value of development of permanent intestinal failure two years post intestinal resection [15].

## 3. Enteral Nutrition

While much of the mainstays of nutritional and medical therapies are based on experience with limited evidence-based support, the practice of early EN after bowel resection has been routinely shown to improve the rate of enteral autonomy [16]. The timing, type, and route of enteral nutrition (EN) can all affect the achievement of enteral autonomy. By the same token, the strategy of “gut rest” causes atrophy in the intestinal mucosa even with appropriate parenteral calories [13,16,17]. Due to the above-mentioned factors, and relatively small numbers of patients with heterogeneous conditions, robust large scale trials in nutritional therapeutics are lacking. Some barriers to progression of enteral nutrition as well as diagnostic considerations and treatment strategies are presented in Table 2.

There are few studies describing the optimal choice for EN in infants with IF. However, human milk, when available is generally preferred as it contains growth factors and immunoglobulins that may promote intestinal adaptation [16]. If human milk is not available, amino-acid based formulas are commonly used based on limited data including a small case series that suggested successful weaning from PN after prolonged use of extensively hydrolyzed formula and a retrospective review suggesting shorter duration of PN with use of amino acid formula [18,19]. There is a suggestion of increased risk of gastrointestinal allergic disease in infants with intestinal failure. One large single center review of infants with IF found that 40% had gastrointestinal eosinophilic inflammation, postulated due to abnormalities in intestinal permeability [20].

The route of feeding delivery–oral/bolus or continuous drip–has variable impact on intestinal absorption, each with varying degree of benefit. In children with PN dependent intestinal disease, continuous drip feedings have been shown to improve nutrient absorption, weight gain, as well as faster time to full enteral feedings [21,22]. In contrast, intermittent or bolus feedings more closely mimic normal GI physiology with cyclic variability in gastrointestinal peptides and hormones [23]. Development of oral skills from a developmental–behavioral standpoint must also considered, therefore introduction of small volumes breast milk or formula as soon by mouth as tolerated may help prevent long-term feeding aversion. Given that the over-arching goal is achievement of enteral autonomy, the practice of our intestinal rehabilitation center, utilizing the available evidence, optimizes early enteral nutrition via a continuous route initially, turning off feedings for one hour a few times per day, to provide the same volume by mouth as enteral rate, as early as possible. Over time, as dictated by patient tolerance, a regimen of increased volumes of oral/bolus intermittent feedings with nighttime continuous feedings is instituted.

## 4. Parenteral Nutrition

Parenteral nutrition is the mainstay of therapy in the management of intestinal failure [13]. PN involves a complex recipe of appropriate macronutrients (carbohydrate, protein, lipid), electrolytes and micronutrients delivered through an IV infusion to provide essential nutrition for individuals with intestinal failure. Administration of PN first requires proper assessment of the unique child’s fluid, electrolyte, and energy needs. Ideally, a collaborative approach is needed with many key team-members including a registered dietician to characterize nutritional needs, a nurse with expertise in vascular access devices and use, a pharmacist to direct the safe preparation of PN components, and a supervising physician with expertise in nutrition [13].

Chronic PN therapy is absolutely a life-saving nutritional strategy for this population; however, there are many inherent risks that require constant attention and prophylactic action. Intestinal failure associated liver disease and catheter related blood stream infection (CRBSI) are among the most concerning as they are the major sources of morbidity and mortality in this population.

## 5. Intestinal Failure Associated Liver Disease

Liver disease, particularly cholestasis progressing to life threatening liver dysfunction, is one the limiting factors in the management of IF [24]. Cholestasis is associated with decreased survival in this population with relative risk of mortality estimated at 20 with conjugated bilirubin ≥2.5 mg/dL [25]. While many macronutrients associated with PN have been implicated as the source of the so-called “TPN cholestasis” or “PN associated liver disease”, this in and of itself does not define the spectrum of the problem. The more accurate term, “intestinal failure associated liver disease” (IFALD) represents the myriad of issues that contribute to severe liver dysfunction in the infant receiving chronic parenteral nutrition. Factors related to development of IFALD include prematurity, fasting/lack of enteral or oral feeding resulting in impaired bile flow, alterations in enterohepatic circulation, infection, as well as PN related factors (namely lipid). The latter two, infection and lipid, have emerged as major contributors to morbidity and mortality, and recent efforts in therapeutic strategies have been geared toward preventing infection and minimizing risk associated with lipid emulsion.

## 6. Role of Lipid in IFALD

Most recently, standard soybean oil lipid emulsion (SOLE), has been implicated as major player contributing to IFALD, postulated due to chronic exposure of pro-inflammatory omega-6 fatty acids (FA) and phytosterols, plant derived sterols that are similar in structure to cholesterol [26]. Inflammatory cytokines are generally cholestatic to the neonatal liver [27]. Prior to 2012, the standard recommended SOLE dose for infants receiving PN was 2–3 g/kg/day.

### 6.1. Lipid Minimization Strategy

In 2012, Teitelbaum and colleagues described significant reduction in cholestasis in a cohort of pediatric IF whose lipid dose was restricted to 1 g/kg/day compared to historical cohort treated with standard therapy. A more recent review of 30 pediatric IF patients requiring PN for >3 years utilizing a lipid minimization strategy demonstrated no essential fatty acid deficiency, no need for transplantation, and normalization of biochemical markers of liver disease [28].

### 6.2. Alternative Lipid Source

Given the inflammatory effects noted with soy based lipid emulsion, alternative sources of lipids have been considered. There have been several studies demonstrating the safety of fish oil based solutions and naturally considered as a regimen that could be used in the management of IFALD [29,30,31]. However, while fish oil as monotherapy for lipid infusions have shown biochemical improvements in cholestasis, there has not been corresponding improvements in liver histology [32,33,34]. To answer question whether the benefits of fish oil were seen as a result of newer treatment paradigm of lipid dose reduction, a randomized control trial was designed. This study aimed to prospectively analyze whether a strategy of lipid minimization with standard soy bean oil versus utilization of fish oil based lipid reduced or halted the progression of liver disease. Interestingly, the study had to be terminated prematurely as the overall incidence of cholestasis amongst the recruited subjects [35]. More recently, a combination lipid emulsion of soy (30%, omega-6 FA), coconut (medium chain triglycerides), olive (monounsaturated FA’s) and fish oil (omega-3 FAs), collectively called, SMOF-lipid, (Fresnius Kabi, Bad Homburg, Germany) has emerged as an alternative [26]. This SMOF-lipid combination has been shown to improve biochemical liver function in patients with IFALD [36]. To date, there has been no randomized trials comparing fish oil lipid emulsion to SMOF-lipid but both seem to be associated with positive results at least in terms of biochemical liver disease.

### 6.3. Role of Infection in IF and IFALD

Infection and inflammation negatively contribute to cholestasis in infants. Catheter related blood stream infection is a major cause of morbidity and mortality, and occurs in high frequency in children with IF who require long term vascular access for PN [37]. The incidence of CRBSI in pediatric patients with IF has been reported as high as 80% [38]. Sondheimer and colleagues reported a retrospective review of 42 pediatric IF patients 67% of which were cholestatic. Interestingly, the cholestatic patients were found to be significantly younger at the time of their first infection [39]. Moreover, advanced liver disease is associated with more infections. Among 30 pediatric patients on chronic PN, those with severe liver fibrosis were shown to have more sepsis events beginning at a younger age compared to those cholestatic patients who had only normal or mild hepatic fibrosis [40].

A generally accepted mechanism is that increased intestinal permeability results in bacterial translocation in the enterohepatic circulation, driving hepatic inflammation [27]. Small bowel bacterial overgrowth (SBBO) is a common finding among children with intestinal failure and may increase intestinal permeability. Alterations in intestinal motility and anatomy, resection of the ileocecal valve, and acid suppression are all thought to be contributing factors making SBBO a particular risk in the IF population. Cole and colleagues found that the odds of CRBSI were seven times higher in pediatric IF patients with SBBO as compared to those without [38]. The diagnosis of SBBO is often clinical with symptoms including malnutrition, vomiting, diarrhea, abdominal distension, D-lactic acidosis. More objective diagnosis by culture of direct aspirates of the small intestine is difficult and limited for several reasons: ease/feasibility of testing given the invasive nature of endoscopic sampling (often requiring general anesthesia), sampling error (single site sampled, bacterial overgrowth may be patchy and exist beyond the length of the endoscope), contamination of oropharyngeal flora, current culture techniques unable to capture the large breadth of GI microbes. A non-invasive strategy is utilization of hydrogen breath testing (HBT). Humans do not exhale hydrogen gas during fasting or at rest because anaerobic metabolism does not occur in these conditions. If measurable hydrogen gas is excreted after ingestion of a non-absorbed carbohydrate, this is presumed to be from the metabolism of the carbohydrate by anaerobic bacteria. Therefore, HBT may indicate the relative quantity and activity of anaerobic bacteria in the intestine. However, these breath tests are difficult to administer in pediatric IF patients due to difficulty of breath collection, rapid intestinal transit time, and interpretation of results. There remains a high degree of false-positive and false-negative results [41].

Cyclical use of non-absorbable antibiotics is the mainstay of therapy for SBBO in most intestinal rehabilitation centers as it often recurs [13,41,42]. However, the optimal management of SBBO is unclear given limited data in children with IF. Typically antibiotics with poor enteral absorption effective against gram negative and anaerobes are utilized. There is suggestion of link between acid suppression and GI infection (presumably including SBBO), suggesting that judicious use or avoidance of acid suppression in setting of SBBO [13,43]. Use of probiotics is typically not recommended in the pediatric IF population as there have been published reports of bacteremia associated with probiotic supplementation [44,45]. In one case, gel electrophoresis of the bacterial isolate from the blood was an exact match of the probiotic that the child was taking; suggesting that the mechanism of sepsis was via bacterial translocation across the gastrointestinal tract [45].

## 7. Medical Therapies

In addition to the antimicrobial strategies used to treat SBBO, other pharmacologic agents with various site of action are part of the arsenal used in the management strategy of IF.

### 7.1. Ethanol Lock Therapy

Ethanol has both antimicrobial and anti-fibrinolytic properties that make it an ideal use as a ‘lock’ solution to prevent infection. 70% ethanol is instilled into the catheter during intervals between use (the so-called ‘ethanol lock’). A meta-analysis of four observational studies utilizing ethanol lock therapy (ELT) in pediatric IF patients, found a reduction of 7.67 CRBSIs per 1000 catheter days [46]. A criticism of ELT is that it is associated with more line complications (line breakage and occlusion). However, a published single center experience did not observe differences in central line complications when on or off ELT [47]. ELT prophylaxis strategy is used on all patients >10 kg enrolled in our center’s intestinal rehabilitation program.

### 7.2. Acid Suppression

While acid suppression is not recommended in the setting of significant SBBO, there is a role for its use in the treatment of gastric acid hypersecretion that occurs post-operatively after bowel resection. Healthy individuals secrete approximately 750 mL/day of gastric acid. Following intestinal resection, there can be a temporary marked increase in gastric acid secretion, up to 4.1 L/day [48]. Proton pump inhibitors or histamine type 2 receptor antagonists are commonly used [42].

### 7.3. Anti-Diarrheal

Significant diarrhea can often impact escalation in enteral nutrition. Diarrhea may be due to accelerated small bowel transit time that results from abnormal anatomy and loss of the so-called “ileal brake”, the inhibitor of intestinal motility normally released from the distal GI tract [49]. Treatment of diarrhea related to rapid transit is often via opioid receptor agonists that inhibit contraction of intestinal smooth muscles [48].

Bile salt malabsorption, or ‘choleretic’ diarrhea, is another important cause of diarrhea in the IF patient. Bile salts are typically absorbed in the distal small intestine. With distal ileal resection with colon in continuity, these bile salts now spill over into the colon. The colonic bacteria deconjugate the bile salts to free bile acids that in turn stimulate movement of chloride and water into the colon. This diarrhea may be treated with bile acid-binding resins (ex., cholestyramine). These resins form insoluble complexes with bile salts which are then excreted in the stool. However, in patients with significant distal small resections, there may be a relative deficiency in bile acids, in this case, the bile acid binding resin may exacerbate fat malabsorption and should be avoided [48].

### 7.4. Prokinetics

Intestinal dysmotility is a contributing factor to enteral feeding intolerance in a number of IF patients, particularly in gastroschisis, intestinal atresia and NEC, where ischemic injury to the enteric nervous symptoms and reparative changes result in alteration in normal motility patterns [49]. Though data is limited, antibiotics including erythromycin (via augmentation of motilin receptors in the antrum) and amoxicillin-clavulanic acid (via propogation of phase III migrating motor complexes) have been used for prokinetic effects [50].

## 8. Surgical Issues and Therapeutic Strategies

### 8.1. Vascular Access

Given the prolonged need for intravenous access for parenteral nutrition, preservation of vascular access sites is crucial. The majority of children in the United States receiving home parenteral nutrition therapy have a tunneled catheter. Again, prevention of CRBSI is of paramount importance to assure maintenance of these vascular access sites.

### 8.2. Intestinal Lengthening Procedures

Surgical strategies have been developed to facilitate tapering dilated bowel and lengthening residual bowel to promote absorption, enhance motility, and limit bacterial overgrowth. The first of these approaches, the Bianchi operation, was first described in 1980. More recently, the STEP procedure (serial transverse enteroplasty), a less technically difficult operation, was developed. A report from the International STEP registry noted that 48 of 97 patients followed over time achieved complete enteral autonomy.

### 8.3. Intestinal Transplantation

Children with intestinal failure may be considered for intestinal or multivisceral transplant for severe progressive IFALD, loss of venous access, recurrent life threatening central line infections, or no chance at enteral autonomy due to anatomic factors. Based on most recent published data from the Scientific Registry of Transplant Recipients, as of 2016 the five-year survival rate for pediatric intestine recipients was 68%, though five-year graft survival for children <age 18 is <60%. The overall number of intestinal transplants has declined since 2008 [51].

## 9. Emerging Therapies

There is ongoing study into other therapeutic options including those that may optimize nutrient absorption and treat IFALD in this patient population. Improving intestinal adaptation via GI hormonal therapy is one promising avenue. Teduglutide, an analog of glucagon-like peptide-2 (GLP-2), is approved for use in adults with SBS. GLP-2 is secreted by enteroendcrine cells in the distal ileum and induces small intestinal epithelial proliferation. In adult studies, Teduglutide has been shown to decrease intestinal malabsorption, increase villous height and crypt depth as well as decrease the need for parenteral nutrition [52,53]. There are recent data suggesting some benefits in children, trials are ongoing. A small open label clinical trial in children with short bowel syndrome revealed that teduglutide was well tolerated with a trend toward reduction in PN requirements [54]. It is hypothesized that altered anatomy short bowel syndrome, disrupts normal enterohepatic circulation of bile acids. A novel ambulatory neonatal piglet model of SBS and PNALD shows promise in use of enteral bile acid therapy to both improve intestinal proliferation and reverse cholestasis and steatosis associated with IFALD [55].

## 10. Conclusions

The care of patients with pediatric intestinal failure requires a multifaceted approach with considerations of nutritional, medical, and surgical needs. A formal multidisciplinary approach by dedicated intestinal rehabilitation teams has demonstrated improved outcomes [56]. Improved care has resulted in decreased need for intestinal transplantation. Future treatment modalities will likely be related to varied approaches to parenteral and enteral nutrition, characterization, and treatment of dysmotility; as well as utilization of hormonal and other endogenous medical therapies to improve intestinal proliferation and treat liver disease. Development of reliable biomarkers to assess the progression of GI function is necessary to aid with ongoing management strategies and long-term treatment decisions. Increased understanding of the gut microbiome in the setting of altered anatomy with practical evaluation modalities and treatment strategies will be useful in the management of serious infectious sequealae associated with intestinal failure. Multicenter trials are needed to improve the quality of evidence to support therapeutic decisions.

## Figures and Tables

**Table 1:** Most Common Etiologies of Peidatric Intestinal Failure.

Most Common Etiologies of Pediatric Intestinal Failure
Short bowel syndrome (Necrotizing enterocolitis, Intestinal atresias, Mid-gut volvulus, Long segment Hirschprung’s disease) Necrotizing enterocolitis
Intestinal atresias
Gastroschisis
Dysmotility

**Table 2:** Signs of enteral feeding intolerance, considerations, diagnostic/therapeutic strategies.

Symptom or Sign	Considerations	Evaluation and Treatment Strategies
Vomiting	Milk protein intolerance	Hydrolyzed or amino acid formula
	Volume sensitivity	Continuous feedings
	Gastric acid hypersecretion	Acid suppression agents
	Obstruction	Contrast study, surgical evaluation
Diarrhea	Formula intolerance	Amino acid formula
	Bacterial overgrowth	Enteral antibiotic
	Enterocolitis	Metronidazole
	Bile acid malabsorption	Cholestyramine
Constipation	Obstruction	Plain films, contrast study, motility evaluation
	Dysmotility including Hirschsprung’s disease	
Abdominal distension	Obstruction	Contrast study, surgical evaluation
Poor growth	Colon in discontinuity? Severe colonic resection?	Check urine sodium; if low, give/increase supplemental sodium
Recurrent sepsis	Inadequate line care	Assure best practices by family, home health care providers
	Bacterial translocation	Treat bacterial overgrowth
